# Stigma respecified: Investigating HIV stigma as an interactional phenomenon

**DOI:** 10.1111/jep.13724

**Published:** 2022-06-16

**Authors:** Phil Hutchinson

**Affiliations:** ^1^ Department of Psychology, Faculty of Health and Education Manchester Metropolitan University Manchester UK

**Keywords:** medical ethics, philosophy of medicine

## Abstract

In this paper, I discuss stigma, understood as a category which includes acknowledged, enacted degradation, discreditation and discrimination. My discussion begins with an analysis of HIV stigma, as discussed in a social media post on Twitter. I then analyse a fictionalized clinical stigma scenario. These two analyses are undertaken to highlight aspects of the conceptual anatomy and interactional dynamics of stigma and by extension shame. Brief social media declarations and short, fictionalized clinical interactions are rich with information which helps us understand how stigma—degradation, discreditation and discrimination—is operationalized in interaction.

## 1 INTRODUCTION

It's Sunday morning at the end of October 2021 and I idly flick through my Twitter feed. A Tweet from the director of aidsmap, HIV activist and campaigner Matthew Hodson catches my eye.

By being open, proud, brazen even about living with #HIV, we dispel the fear and ignorance that leads to stigma.

#RespectMyHIV

No shame in HIV.

This tweet caught my attention because in a few words it captures so much, and in unpacking it we can see the elucidatory work being done in the three short sentences that comprise the tweet.

So, first, and perhaps most obviously, the tweet testifies to the persistence of shame and stigma as prominent negative factors which people living with HIV must contend with, negotiate and combat simply in virtue of their HIV status. Even now, when the medical treatment of HIV means a person living with the virus can live a full, healthy life without fear of transmitting the virus to others, shame and stigma too often accompany an HIV diagnosis and a life lived with the virus.

The tweet also testifies to the link between stigma and shame, something that is, oddly, not widely in evidence in the extensive academic literature on HIV stigma, which often exclusively focuses on stigma. However, this relationship between shame and stigma is important.^1^, ^2^ While the link with shame has been largely overlooked in discussions of stigma in HIV research, philosophers and psychologists who work on emotions provide a way of understanding this relationship by talking of emotions having (intentional) objects, which are partly constitutive of an emotion's meaning. Another way of putting this is as follows: how an emotion is specified is informed by what the emotion is about or directed at; so, in the case of stigma‐shame, one's shame is about or directed at one's experience of stigma. Put another way, it is part of what it means to feel this shame to have acknowledged oneself *degraded*, *discredited* or *discriminated* against and these are the phenomena which are brought together by the category of stigma. So, the relationship between stigma and shame here is *internal*. What this means is that where shame has stigma—degradation, discreditation or discrimination—as its object, as what it is about, then that stigma is part of what shame is; it becomes part of the meaning, part of the content, of one's shame. To lay it out: the term ‘stigma’ denotes a category of acknowledged, enacted discreditation, degradation and discrimination, and these phenomena are internal to the meaning of the shame one experiences when stigmatized.*

In testifying to the relationship between shame and stigma, Matthew Hodson's tweet goes further; his tweet is making a declaration: he is/will be, ‘open, proud and brazen even’ about his HIV status and this will serve to deny shame a foothold. Pride is the antonym of shame, and Matthew Hodson is asserting pride in his status as a man living with HIV. Shame is characteristically associated with withdrawal^3^ and closing oneself off from the social world, and Matthew Hodson is declaring openness, and doing so in a public social media post. Finally, we have the commitment to be ‘brazen’, a word for which *shamelessness* is often listed as a synonym. So, Matthew is declaring that he will resist attempts to discredit or degrade him based on his HIV status; he will refuse to acknowledge discriminatory interactions as reflective of his status as a person. Matthew is asserting his agency, his commitment to exercise control and affirm his worth.

One way of unpacking this is to observe that social phenomena, such as discreditation, degradation and discrimination are interactionally produced and that members of situations, of social or interactional contextures, are those who, through their interactional work, produce the phenomenon. Matthew Hodson is asserting that he will resist taking a role in the production of stigma, by being open, proud and brazen about his HIV.

This leads to a third observation that we can make about the tweet. In addition to highlighting for us the internal relationship between stigma and shame and his commitment to reject stigma and shame by affirming his pride, openness and brazenness, Matthew Hodson's short tweet also testifies to how stigma, as a category term for acknowledged, enacted discreditation, degradation and discrimination, is not about discrediting or degrading *attributes*, where the attribute is understood to have fixed or invariant (discrediting or degrading) meaning. Rather, the attributes which are the locus of shame and stigma are *indexical*; that is to say, their *meaning* is connected to, is indexed to, how they are operationalized in particular contextures, in interactionally constituted situations, in which one participates. HIV isn't invariantly degrading or discrediting, it is so, where it is, only when it has been operationalised as such. Matthew Hodson recognizes he has input into these interactionally produced contextures and therefore to the meaning his HIV has. He is declaring that he will challenge attempts to depict his HIV as discrediting, degrading or as grounds for discrimination and will proactively proclaim his HIV as a source of pride and as status‐affirming.

HIV is stigmatizing, when it is, because of how it is oriented, and how its meaning is locally produced and fixed, in specific interactional situations. These situations have the character of Gestalt contextures, where the meaning of the situation is constituted by the actions of those comprising the situation, while those actions have the sense they do as parts, or constituents, of the situation.† One goal for us, as stigma researchers, is to recover how, and in what ways, HIV is operationalized to be stigmatizing and elicit shame. What needs to be in place for that to happen? In seeking to do this, we could do much worse than learn from those such as Matthew Hodson, by studying the day‐to‐day work of resistance, reframing and status affirmation they undertake.

The final point contained in Matthew Hodson's tweet that I shall discuss is the claim that ‘fear and ignorance lead to stigma’. While this is another important insight contained in the tweet, I want to use it to also make a case for exercising caution. Undoubtedly, some of the degrading, discrediting and discriminatory attitudes and behaviour directed at a person living with HIV are based on fear and ignorance and it is therefore important to take steps to combat this. However, it is also important to recognize that such a strategy alone will not eradicate stigma because there are instances of degrading, discrediting and discriminatory behaviour which,

a. aren't intentional or deliberate. For example, there are cases where the stigmatiser does not intend to and nor are they deliberately stigmatizing; indeed, a person might act to degrade another yet be sincerely shocked and remorseful in finding that their actions were experienced as degrading, and
b. even where the stigmatization is intentional or deliberate it might not be based on ignorance of facts but rather based on a value judgement made with disregard, ambivalence or contempt for the facts about HIV prognosis and transmissibility. Status‐degrading interactions might be interactions driven by (negative) value judgements of one party to the interactions, and value judgements can be and often are formed with indifference to facts. Motives for discrimination might turn out to be fact‐free, prejudice‐based hostility toward people with different lifestyles, taking sides in a culture war, the enactment of moral mythologies, convenient ‘sticks’ to beat people with to obtain a social advantage, tribalism, opportunistic ways of expressing anger, attempts to gain advantage or power over an interlocutor, and such like.

So, while combatting ignorance of the facts about HIV is both important and will go some way towards reducing HIV stigma, eradicating ignorance will *not* eradicate stigma. Discrimination is not exclusively based on ignorance of facts because facts often don't feature at all in discrimination. Discrimination is often based on a fact‐free negative *evaluation*. Indeed, in some cases, even where putative facts (or factoids) do feature in an attempt to justify degrading or discrediting behaviour they do so in a way which is secondary to, or in service of, the (priorly embraced) values; so, the (negative) evaluation frames, filters or distorts the (putative) facts that are appealed to. Put another way, even where ignorance is a feature of stigma, that ignorance can be *willful* ignorance motivated by values. To address willful ignorance we need to address it at the level of the motivating values that underpin that will to ignorance.

I began with Matthew Hodson's tweet not only because in a few short sentences it captures a number of the issues that I want to discuss but also to show that these distinctions are there to be found in the conversations, interactions and declarations one finds ‘in the wild’. We do not need academic theories that define stigma, we need to look carefully at how people who are subject to stigma negotiate, combat, communicate about, challenge, avoid and make witnessable their experiences of discreditation, degradation and discrimination. We don't need uniquely academic analyses of structural stigma, we need rather see how structural discreditation, degradation, discrimination, bias and so on are operationalized and made manifest in interaction. Furthermore, we need (much) more than surveys of stigma, which *at best* give us a very rough idea of the prevalence of stigma in society. These are of little use. We need to understand the conceptual anatomy and the interactional dynamics of stigma, by focussing on interaction.

In what follows, I will analyse a stigma scenario, with a view to exploring the interactional dynamics and conceptual anatomy of stigma. Before I lay out the example and conduct the analysis I will say a little about the method I am employing.

## 2 EXAMPLES, CASE STUDIES AND SITUATION ANALYSIS

My practice in what follows will take the form of *Situation Analysis*, an approach which Doug Hardman and I are developing together^7^ which is derived from the philosophical method of Frank Ebersole.^8^, ^9^, ^10^ I take this to be a companion to the philosophy‐as‐case‐studies or field‐philosophy of Harold Garfinkel^4^, ^5^ and its development at the level of conversation in the work of Harvey Sacks and his collaborators.^11^ Here, the examples and case studies are not employed, as they are in experimental social psychology or experimental philosophy, as empirical data or evidence, but are instead treated as aids to the imagination, ways of bringing to light unseen aspects and meaning relations, drawing attention to particulars which might otherwise have been overlooked, and as tutorial devices which help us grasp the conceptual anatomy and interactional dynamics of phenomena. The examples and case studies can be 'found', imagined and fictionalised.

The purpose of pursuing this method is to find the sense in certain words and actions as part of a situation when that sense might be in question. This is an art, not a science; our examples are not there to serve as evidence to settle matters, much less to prove a hypothesis, but as resources for reflection and as ‘objects of comparison’.^6^,^sec.130^ We find sense by reflection on the situation—the Gestalt contexture—that might render the action or claim intelligible in the context in which it is undertaken or made while recognizing that the identity of the situation is at the same time a product of those same activities. We can, therefore, depict *Situation Analysis* as follows: we take examples of activities, including language use, and explore contexts, understood as interactionally produced Gestalt contextures, in which those examples would have sense *and* the sense they would have in those Gestalt contextures. Wittgenstein's language‐games are Gestalt contextures, which are both produced by what we say and do but which in turn confer upon our doings and sayings the sense they have in this contexture/language‐game. Our task in working with our examples is to find the sense in the situation, by identifying the Gestalt contexture(s), or the language‐game(s) being played.

## 3 OUR EXAMPLE/CASE STUDY

The following example is a *fictionalized* example.

A patient, Karolina, visits the doctor for an appointment for breathing problems. The patient is not new to this Health Centre, but today's doctor, the GP, is new to this patient. The first minute of the consultation has gone without anything of note occurring. Following initial greetings, the exchange is polite and unremarkable. Both patient and doctor seem to be conversing naturally, as would be expected in such a setting, and a rapport seems to be developing. The situation is mundanely social—two people who've not met before are interacting politely so as to communicate effectively. They take turns in speaking, questions elicit answers, and smiles, nods and audible ‘mmmm's are used to indicate each is listening to the other, without interrupting. In addition to being mundanely social, the interaction has some institutionally specific features—it is taking place in a health centre during clinic hours, in the presence of paraphernalia that exhibits this: the room contains an examination table, clinical waste bins, the doctor is wearing an NHS lanyard and ID card. Moreover, the doctor is sat in front of a PC, and, as the patient enters, invites them to take a seat on a chair adjacent to the PC and so on. In addition to these situational features, the conversation in and of itself exhibits the difference in roles, or membership categories,^12^, ^13^ of the two participants. The conversation, in its unfolding, indicates that one person has experiential authority: that is, the patient is the authority on the experience of the symptoms, which is exhibited in the form the conversation has, the types of questions the patient asks and the type of questions asked of her. Equally, the conversation indicates that the other party has medical authority: that is, the doctor has medical training which confers authority in this situation on how to interpret the patient's reported experience of the symptoms and arrive at a diagnosis, and this is exhibited in the conversation by the kinds of question the doctor asks, the kinds of questions asked of her by the patient and the kinds of answers the patient provides. The roles, or membership categories, are co‐constituted by and exhibited in the interaction. This segues into our final observation about the situation, and that is that there is a witnessable goal‐ or achievement‐orientation, observable in their interaction—the patient exhibits that they want a diagnosis and treatment to relieve their symptoms; they also want to understand what it is they are experiencing. The doctor wants to provide this, in the time allotted for a consultation. We read this off the institutional context: the patient is attending an appointment at a GP surgery, but we can also recover this information by close attention to the conversational exchange. Such as the doctor's greeting including the question 'so what seems to be the problem today' and so on.

Two minutes into the consultation, things change and understanding why they do so and making sense of the actions that both immediately precede and take place after proceedings have taken this turn will serve as our topic of investigation.

After the opening greetings and a couple of minutes or so of polite interaction, the doctor, smiling, glances at the screen, and then begins to turn as if to re‐engage in conversation with Karolina before appearing to hesitate and returning focus to the screen. The doctor's smile drops momentarily, and she seems to be distracted or absorbed by what she is looking at on the screen, before re‐initiating the turning in her chair to face Karolina. Concurrently, Karolina seemed to notice the doctor was about to re‐engage and was therefore preparing for re‐engagement herself, as the doctor began to turn; only this re‐engagement action was discontinued as she saw the doctor break off to return to the screen. As the doctor turns from the screen the second time and re‐initiates the conversation, she once again faces Karolina, smiles and asks ‘do you mind if I take a look at your throat and listen to your breathing?’, while, at the same time, putting on a pair of latex gloves.

At this moment, Karolina's posture changes, her smile turns to a frown, and her posture becomes what would usually be referred to as ‘withdrawn’ (a slight lean and turn away from the doctor) and ‘closed’ (eye contact avoided, arms folded), and she furrows her brow, frowns and trembling hands are visible as she unfolds her arms. Karolina remarks, ‘Why are you doing that?’. … She waits a few seconds. The doctor stutters. Their rapport is broken; for a moment the doctor hesitates, pausing the putting on of the latex gloves, stuttering and not completing her attempt at a verbal response. Before the doctor can compose a response, Karolina picks up her bag and makes to leave the consulting room. The doctor begins to ask ‘is everything…’ but, interrupting, Karolina remarks ‘what do you think?’ and leaves the consulting room.

## 4 SEEING AN ACTION UNDER AN ASPECT: THE MEANING OF PUTTING ON THE GLOVES

The mundane sociality and convivial rapport observable in the first 2 min gives way to discordance and trouble. We can observe, from the example, that Karolina's anger and the social discord were immediately preceded by the doctor's attention being seemingly grabbed by something on the screen, this causing her to break off and initiated re‐engagement and then by her beginning to put on the latex gloves. What comes after is *discordance*, because while the doctor seems to proceed as if there is no change in circumstance, or Gestalt contexture (until she registers and reacts to Karolina's reaction), Karolina, from this moment on, acts in a way which suggests circumstances (the Gestalt contexture) have changed for her. Indeed, we might read Karolina's body language and behaviour as indicative of shame: closure and withdrawal. Let us try to unpack this.

So, we have three actions:

1. an aborted re‐engagement in the ongoing conversation by the doctor,
2. a visible (to Karolina) case of the doctor's attention being grabbed by something on the screen, and
3. the doctor beginning to put on the latex gloves while requesting the patient's consent for an examination.

It seems safe to make the following observations about these three actions: they seem mundane, routine and unremarkable to the doctor at the moment of enaction. For the doctor, these actions have nothing about them to set them apart from their other contributions to the interaction before that point. The sense they have for the doctor at the point of enaction is as routine acts in an unremarkable unfolding of a routine consultation. For Karolina, one or more of these actions seems to be the object of her shame and anger, and, therefore, the emergent discord, in that they sequentially precede Karolina's reaction in withdrawing. What this means is that one or more of the actions has a different sense for Karolina than for the doctor. It is *that* sense which makes that/those actions objects for Karolina's emotional response. How might the same actions have different senses? Well, this is where we might invoke the idea of praxiological Gestalt contextures. There are two things:

1. The relationship between the sense the actions have and the sense of the contexture of the situation as a whole and
2. The sequential production or weaving of the contexture, or ongoing establishing of the rules of the language‐game, through the moves the participants make.

For the doctor, her actions have sense in the contexture that is a *mundane, routine consultation*, until Karolina indicates it isn't and discordance emerges in the proceedings. For Karolina, the doctor's broken‐off re‐engagement and absorbed focus on the screen indicated a move to a *different contexture* in which the actions are now reframed by her HIV status; this sequence of actions was experienced by Karolina almost like the familiar opening to a melody. The contexture, for Karolina, now splits off from one of a routine consultation and becomes one of HIV‐related degradation. Karolina recognizes such interactions; she's seen them before. The doctor's return to and extended focus on the screen was seen by Karolina as being the moment the doctor registered her HIV status in her medical records; this action is followed sequentially by the doctor putting on the latex gloves. This is a routine action for a clinician who is about to examine a patient, but this patient had disclosed their status as a person living with HIV on a previous visit while also being unaware of the routine and required practice of glove wearing when examining all patients in a clinical setting. The patient saw the action as related to or directed toward their HIV status and felt shame and anger in response.

What can we learn from this? Well, if we take degrading someone to be unethical or something best avoided because emotionally harmful, counterproductive to good care and compassionate interaction, then what we see here is that ethical or emotional harm need not be intentional and need not be constituted by the violation of a moral rule. The patient sees the action under a specific aspect, given their HIV status, what they know about the stigma associated with HIV and what they know about the persistence of ignorance about HIV transmission (that many people, even some GPs, aren't aware that fully suppressed HIV cannot be transmitted). Seeing the action sequence as Karolina does, leads her to *see* the glove‐wearing *as* being because the doctor knows she is living with HIV and the doctor is, therefore, out of ignorance and prejudice, seeking to ‘protect’ herself from the patient. The patient *sees* the putting on of the gloves under this aspect, *as* having this sense. For Karolina, the doctor's broken‐off re‐engagement and absorbed focus on the screen indicated a move to a different contexture in which the actions are now reframed by her HIV status; this sequence of actions was experienced by Karolina almost like the familiar opening to a melody, where each note is heard as part of the melody and as pregnant with the notes which follow. This is a familiar ‘tune’ for Karolina, one she has heard many times before.

The patient *sees* the doctor putting on gloves *as* degrading her; the sequence of three actions that culminate in this relates to a newly established Gestalt contexture, of establishing new rules and therefore a new language‐game, where the action of putting on gloves, in this language‐game, is seen as ‘putting‐on‐gloves‐as‐protection‐from‐you‐because‐you‐have‐HIV’.

The doctor *sees* the putting on of the gloves *as* a routine part of her own medical practice: she is simply putting‐on‐gloves‐for‐routine‐examination. The same behaviour has a different sense; there is discord. It can be tempting here to ask which comes first the contexture or the act, the language‐game or the action as a move within it, but this is a misguided question. Seeing the action as a precautionary act directed at this patient's HIV status rather than as a routine act is also to establish a new contexture, *at the same time*.

The question as to who has seen the action correctly here is academic, because in the world the harm is done. This would be like saying that seeing the Jastrow duck‐rabbit picture‡ as a duck and not as a rabbit is the correct way of seeing it because you have ducks at home, had duck eggs for breakfast and have never seen line drawings of rabbits before. Sure, these serve to clarify to us *why* you see it as a duck, but they do not carry over to general claims about the *right* way to see the picture. Put another way, the question of intent is irrelevant to the question of the reality or objectivity of acknowledged, enacted degradation.

We could go into more detail about this example, we could talk about how we might discuss questions about such things as the responsibility of the doctor to anticipate and seek to block the emergence of this contexture by always explaining why they are putting on gloves before doing so, or by giving an account as to what took their attention on the screen. If we had AV data, perhaps we could do even more work (though not necessarily). However, the point I want to make is that for something to be an *enacted degradation* the action needs to have that sense and be *acknowledged* as such by the person experiencing the degradation. The sense emerges from the internal, or meaning, relationship holding and being acknowledged between the identity of the specific action, which is the doctor putting on gloves as protection against this patient's HIV, and the identity of the Gestalt contexture, as an interaction in which one's HIV status is the motive for behaviour. This relationship is established as the actions are, sequentially, reconstituting the Gestalt and the Gestalt is the pattern or form of which those actions are seen as parts. What we see in acknowledged, enacted degradation, discreditation and discrimination, where the degrading act was not deliberate or intended, can be understood by our seeing the divergence of contextures take place in the interaction. To see the emergence of the contexture which enables the degradation or discreditation, one needs to see it from the perspective, under the same aspect as, the person degraded and discredited, to the extent that this is contexture forming. In terms of our talk of this in terms of meaning, you must be alive to the meaning relations that are in play for the person who has been degraded or discredited. This certainly demands you must see the situation via the concepts available to the participants. It also might mean that you need to have some degree of unique adequacy,^5^, ^14^ such that you see the internal relation between, for example, HIV and status‐degradation. If you don't, if, for example, you think HIV is just a virus like the common cold and do not know the grammatical relation between HIV and fear, generated over decades by public health messaging that employed fear tactics, then you will be unable to see this relationship in situations like we've discussed here.

## 5 CONCLUSION: THERE IS NOTHING OUTSIDE THE INTERACTION

Whether one thinks the term ‘stigma’ is useful or not, whether one thinks it is a term that can usefully specify a topic for analysis or not are questions I am happy to remain agnostic about. The important point is, rather, where we look if we want to understand how degradation, discreditation and discrimination get done and what ‘mechanisms’ or interactional dynamics we might observe in their accomplishment. I have suggested that the accomplishment can be made sense of by seeing a divergence of contextures in interaction, which facilitate the seeing of certain actions as degrading, where they don't, at the point of enaction, have this sense for the person carrying out those actions or a third‐party observer. We might say, as part of this new contexture, the degradation is a *fait accompli*. It's not a phenomenal overlay or interpretation it is a social fact, a phenomenon.

The point I want to make is this: such that we experience the social world, we experience social phenomena—that is, things that are constituted by people's actions while not being reducible to those (individuated) actions. Stigma is undoubtedly a useful analytic category, but it is so because it serves certain formal analytic purposes for the analyst. Stigma as an analytic term categorizes types of degrading, discrediting and discriminatory phenomena so we can say something general about those phenomena at the level of the category. There's nothing necessarily wrong with this, so long as we are clear that this is what we are doing when employing the term stigma. However, if you want to understand the interactional dynamics of degradation, discreditation and discrimination and how these are enacted in social situations, then you need to do so by identifying the social phenomena as they are experienced by the person who is being degraded, discredited or discriminated against.

What we generally refer to as stigma is a complex category of phenomena and we need to understand the interactive dynamics of those phenomena if we are to address stigma. In many cases, self‐degradation, anticipated discreditation and nonintentional degradation are a big part of the problem and serve as the scaffold or support for the more commonly discussed degrading, discrediting and discriminatory acts, which are, perhaps, morally motivated, and intentional.

I chose as my example in this paper a degradation in which there was no intention to degrade and no ignorance about HIV on the part of the person who performed the degrading act, so as to emphasize the extent to which such instances of stigma are to be found and might even be pervasive. This should help us guard against the widely‐held view that stigma—degradation, discreditation and discrimination—is (always) caused by ignorance and the solution to, say HIV stigma, will be found solely in education campaigns.

## ACKNOWLEDGEMENTS

This paper was presented at the *Shame, Health and Lived Experience* workshop organized by the *Shame and Medicine Project* and hosted by the *Centre for Subjectivity Research* at the University of Copenhagen, Denmark, May 2022. Earlier draft material was discussed at the *Manchester Ethnomethodology Reading Group*, in May 2021 and in a *National Centre for Research Methods* (NCRM) course I delivered on Situation Analysis, in February 2022. Thanks to the participants in those three fora for some insightful comments, questions and discussions that helped improve the arguments. Thanks also for written comments and extended discussions on this paper and on my work on interactional stigma more generally to Loreen Chikwira, Khadijah Diskin, Luna Dolezal, Clemens Eisenmann, John R. E. Lee, John A. Rooke and Sven Schaepkens. Thanks in particular to Matthew Hodson who graciously said he was happy for me to use his tweet in the way I do.

## CONFLICT OF INTEREST

The author declares no conflict of interest.

## Data Availability

Data sharing not applicable to this article as no datasets were generated or analysed during the current study.

## Footnotes

* This is a point about the *relationship* between an emotion, such as shame, and its object (what the emotion is about), such as (but not exclusively) stigma; I am not seeking to provide a definition of shame, much less define shame as always having stigma as it's object. Stigma features prominently as the object of shame, but it is not exclusively the object of shame. Where stigma is the object of shame, the relation between shame and stigma is not external (e.g., causal) but internal (meaning). What is being argued is that where shame has stigma as its object, then that stigma—the discrimination, discreditation or degradation—is part of the meaning that shame has for the person experiencing it.

† This idea can be traced to Harold Garfinkel's work,^4^, ^5^ in which he conceives of social phenomena as interactional or praxiological Gestalts. The same logic can be discerned in Ludwig Wittgenstein's idea of language‐games.^6^ The key point is that there is a kind of synergy between the identity or meaning of the whole and the meaning of the constituent acts or utterances. So, we make the basic contextual point that my actions might be stigmatising in one situation but not in another and then show that the context, the situation, is itself a context*ure*, which is co‐constituted by the inter*actions* of the members of that contexture.

‡

## References

[1] Hutchinson P , Dhairyawan R . Shame and HIV strategies for addressing the negative impact shame has on public health and diagnosis and treatment of HIV. Bioethics. 2017;32(1):68‐76. 10.1111/bioe.12378 28833363 PMC5763398

[2] Hutchinson P , Dhairyawan R . Shame, stigma, HIV: philosophical reflections. Med Humanit. 2017;43(4):225‐230. http://mh.bmj.com/content/medhum/43/4/225.full.pdf 28790137 10.1136/medhum-2016-011179 PMC5739830

[3] de Hooge IE , Breugelmans SM , Wagemans FMA , Zeelenberg M . The social side of shame: approach versus withdrawal. Cogn Emot. 2018;32(8):1671‐1677. 10.1080/02699931.2017.1422696 29303420

[4] Garfinkel H . Studies in Ethnomethodology. Prentice‐Hall; 1967.

[5] Garfinkel H . Ethnomethodology's Program: Working Out Durkheim's Aphorism. In: Rawls AW , ed. Rowman & Littlefield Publishers; 2002.

[6] Wittgenstein L . Hacker PMS , Schulte J , eds. Philosophical Investigations. 4th ed. Wiley‐Blackwell; 2009.

[7] Hardman D , Hutchinson P. Where the ethical action is. J Med Ethics . 2021. Published online. 1‐4. 10.1136/medethics-2021-107925 34952893

[8] Ebersole FB . Things We Know: Fourteen Essays on the Problem of Knowledge. 2nd ed. Xlibris; 2002.

[9] Ebersole FB . Meaning and Saying: Essays in the Philosophy of Language. 2nd ed. Xlibris; 2002.

[10] Ebersole FB . Language and Perception: Essays in the Philosophy of Language. 2nd ed. Xlibris; 2002.

[11] Sacks H , Jefferson G , ed. Lectures on Conversation. Vol 1‐2. Blackwell; 1995. 10.1002/9781444328301

[12] Fitzgerald R , Housley W , eds. Advances in Membership Categorisation Analysis. SAGE Publications Ltd; 2015.

[13] Schegloff EA . A tutorial on membership categorization. J Pragmat. 2007;39(3):462‐482. 10.1016/j.pragma.2006.07.007

[14] Rooke CN , Rooke JA . An introduction to unique adequacy. Nurse Res. 2015;22(6):35‐39. 10.7748/nr.22.6.35.e1342 26168812

